# Can letrozole plus HMG protocol improve pregnancy outcomes in frozen-thawed embryo transfer? An RCT

**Published:** 2017-02

**Authors:** Ashraf Aleyasin, Marzieh Aghahosseini, Leili Safdarian, Maryam Noorzadeh, Parvin Fallahi, Zahra Rezaeian, Sedighe Hoseinimosa

**Affiliations:** 1 *Department of Obstetrics and Gynecology, Infertility Unit, Shariati Hospital, Tehran University of Medical Sciences, Tehran, Iran.*; 2 *Fertility and Infertility Center, Shariati Hospital, Tehran, Iran *

**Keywords:** Frozen-thawed embryo transfer, Endometrial preparation, Freeze embryo

## Abstract

**Background:**

There are different methods in endometrial preparation for frozen-thawed embryo transfer (FET).

**Objective:**

The purpose of this study was to compare the live birth rate in the artificial FET protocol (estradiol/ progesterone with GnRH-agonist) with stimulated cycle FET protocol (letrozole plus HMG).

**Materials and Methods:**

This randomized clinical trial included 100 women (18-42 years) randomly assigned to two groups based on Bernoulli distribution. Group I received GnRH agonist [Bucerelin, 500μg subcutaneously] from the previous midlutea lcycle, Then estradiol valerat [2 mg/ daily orally] was started on the second day and was increased until the observation of 8mm endometrial thickness. Finally progesterone [Cyclogest, 800 mg, vaginally] was started. Group II received letrozole on the second day of the cycle for five days, then HMG 75 IU was injected on the7^th^ day. After observing [18 mm folliclhCG10000 IU was injected for ovulation induction. Trans cervical embryo transfer was performed in two groups. The main outcome was the live birth rate. The rate of live birth, implantation, chemical, and clinical pregnancy, abortion, cancellation and endometrial thickness were compared between two groups.

**Results:**

Implantation rate was significantly higher in group I. Live birth rate was slightly increased in group I without significant difference (30% vs. 26%). The rate of chemical and clinical pregnancy was similar in two groups. The abortion rate was lower in letrozole protocol but the difference was not statistically significant. The mean endometrial thickness was not different between two groups.

**Conclusion:**

Letrozole plus HMG method cannot improve pregnancy outcomes in frozen-thawed embryo transfer but it has only one injection compare to daily injections in artificial method.

## Introduction

Frozen thawed embryo transfer (FET) has become a successful technique of Invitro Fertilization (IVF). Nevertheless, FET is a less invasive procedure for patients, reduces cost and time period, increases the cumulative pregnancy rate, and reduces multiple pregnancies (1-5). An important factor in this process is synchronization between the embryo and the endometrial development (6-9). There are different cycle regimens for endometrial preparation: natural cycle (NC-FET), modified natural cycle, stimulated cycle FET, and artificial cycle FET (AC-FET). NC-FET is a simple method, but the variation in timing of ovulation between cycles is its disadvantage. In NC-FET follicle monitoring should be done with several ultrasounds and is expensive. Modified natural cycle is an alternative method in which ovulation induced by drugs such as letrozole, clomiphene citrate or Human Menopausal Gonadotropin (HMG). In AC-FET the endometrium is prepared by estradiol and progesterone hormones with or without GnRH-agonist. There are different studies for comparison of these protocols (10-13).

The purpose of this study was to compare the pregnancy outcomes in AC-FET with the stimulated-FET prtocol for endometrial preparation in frozen thawed embryo transfer.

## Materials and methods

This randomized controlled trial was performed at Shariati Hospital of Tehran University of Medical Sciences, Tehran Iran between February 2014 and February 2016. Inclusion criteria were all women (18-42 yr old) who were undergone endometrial preparation for first frozen embryo transfer. Infertile couples with male infertility undergone testicular sperm extraction (TESE) or percutaneous epididimal sperm aspiration (PESA); severe endometriosis (stage 3 or 4); uterine myoma≥4 cm, and fresh embryo transfer were excluded. The samples (N=100) were divided into two groups randomly based on Bernoulli distribution.

GroupI(AC-FET group, received GnRH-a (Bucerelin, Aventis, Germany) 500μg subcutaneously from the previous midluteal cycle (21^st^ day). Then estradiol valerate (Daroopakhsh, Iran, 2 mg/ daily orally) was started on the second day and was increased until the observation of 8mm endometrial thickness in transvaginal ultrasound. For visualizing endometrial thickness, transvaginal ultrasound (Sonoline G20; Siemens Medical Solutions, California, USA) was performed every 4 days. After the observation of at least 8 mm endometrial thickness, progesterone (Cyclogest, Germany, 800 mg) was started vaginally. After 3 days, about three good quality embryos (blastocyst stage) were transferred on day 16-19 by catheter (COOK MEDICAL Embryo Transfer).

GroupII, (stimulated-FET group, received letrozole (Iran hormone, Iran, 5 mg/daily) orally on the second day of the cycle for five days. Then HMG (Ferring, Germany, 75IU daily) was injected on the7^th^ day. After the observation of 18mm follicle in trans vaginal ultrasound, human chorionic gonadotropin (hCG) (Ferring, Germany, 10000IU, and IM) was injected for ovulation induction. About three good quality embryos (blastocyst stage) were transferred on day 16-19 by catheter (Cook Medical Embryo Transfer). The policy to transfer up to 3 embryos in patients was adopted in two groups. The rate of live birth, implantation, chemical, and clinical pregnancy, abortion, and cancellation were compared between two groups. Chemical pregnancy was through serum βhCG analysis 16 days after frozen embryo transfer. Clinical pregnancy was detected by pregnancy sac observation in trans vaginal ultrasound two weeks after positive pregnancy test.

**Ethical consideration**

This study was approved by Ethics Committee of Tehran University of Medical Sciences [Ref. number: 90-04-30-15625-52546]. All participants signed an informed consent for participation in this study.

**Statistical analysis**

All data were analyzed by SPSS software (SPSS, version 21 for windows SPSS Inc., Chicago. IL). Statistical tests were Student's *t*-test and Chi-square test. P<0.05 was significant.

## Results

One hundred women undergone first FET were enrolled in two our study groups (n=50/each). There were no significant differences in age, duration of infertility, and number of transferred embryos between two groups (Table I). Pregnancy outcomes in both groups are shown in table II. There were 18 chemical pregnancies in each group. In group I, from 18 pregnancies, 8 pregnancies were aborted. There were 10 deliveries (20.0%) in group I (3 twins and 1 triplet). In group II, from 18 pregnancies, 6 pregnancies were aborted. There were 12 deliveries (24.0%) in group II (one twin). We had 15 live births (30%) in group І and 13 (26%) in group II.

**Table І:** Comparison of demographic characteristics in two groups

**Characteristics**	**Group** ** І** ** (AC-FET group)** **(n=50)**	**Group ** **П** ** (stimulated-FET group)** **(n=50)**	**p-value** **
Age(years)*	30.10±4.34	30.54±4.11	0.60
Infertility duration (years)*	6.24±*3.58*	7.04±3.47	0.26
Number of transferred embryos*	3.16±0.51	3.19±3.19	0.80

AC-FET: artificial frozen embryo transfer

* Mean±SD

** Independent Student's*t*-test was used

**Table П:** Comparative analysis of pregnancy outcomes

**Characteristic**	**Group ** **І** ** (AC-FET group)**	**Group ** **П** ** (stimulated-FET group)**	**p-value**
Endometrial thickness(mm)*	8.06±0.31	8.08±0.40	0.750
Chemical pregnancy rate	18.50 (36%)	18.50 (36%)	0.582
Clinical pregnancy rate	13.50 (26%)	14.50 (28%)	0.500
Delivery rate	10.50 (20%)	12.50 (24%)	0.454
Live birth rate	15.50 (30%)	13.50 (26%)	0.405
Implantation rate	11.39%	9.8%	0.029
Abortion rate	8.50 (16%)	6.50 (12%)	0.734

Independent Student’s *t*-test was used

Because of having twins live birth is more that clinical pregnancy outcomes.

* Mean±SD

**Figure 1:**
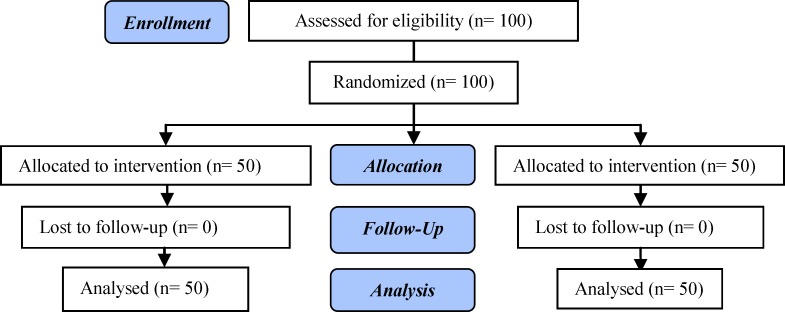
Consort flow chart

## Discussion

In this study, we detected that live birth rate was slightly increased in artificial protocol without significant difference. Although implantation rate was significantly higher in the artificial cycle, chemical pregnancy rate was equal in two groups (36%). In letrozole protocol, the rate of clinical pregnancy was slightly increased and the abortion rate was lower. In this group the delivery rate was higher than artificial protocol (24% vs. 20%) but was not statistically significant. Multiple pregnancies were higher in artificial group than letrozole group, then live birth rate was slightly increased in this group.

In a recent study carried out by Sibai *et al* (2016) in which 94 cycles of letrozole were compared with 96 cycles of hormonal preparation the ongoing pregnancy rate was significantly higher in the letrozole group (47.9% v 32.3%) (p=0.02) but the chemical pregnancy rate was not significantly higher (53.2% v 40.6%) (14). Song-jun Li *et al* reported that the embryo implantation rate, clinical pregnancy rate, and live birth rate of patients in letrozole group was significantly higher than the HRT group (30.4 % vs. 22.8%), (53.2% vs. 44.4%), and (44.6 % vs. 32.5 % respectively) (15). In Huang study, in 689 letrozole FET cycles, the live birth rate was 38.89% and the clinical pregnancy rate was 47% (16).

We found that spontaneous abortion rate was not significantly lower in the letrozole group (12% vs, 16%). This is in agreement with the observation of Sibai *et al *(5.3% v 8.3%)while in Li study this rate was significantly lower than the HRT group (12.0% vs 21.0%) (14, 15). The mean endometrial thickness before transfer did not vary between two groups. In Sibai and colleagues study the mean endometrial thickness in the letrozole group was significantly lower (9.9 mm vs. 9.1 mm) (14). 

In a study carried out by Jouan*et et al *the pregnancy rate per transfer in clomiphene citrate group was higher than AC-FET group (24.3 vs. 20.8) while miscarriage rate was lower (23.2 vs. 29.8) (17). There are several studies about endometrial preparation protocols in FET. Konc* et al* (2010) in a retrospective study compared three cycle regimens: natural, stimulated (HMG/rFSH) and artificial. The pregnancy rate in the natural cycle was 34.9%, in the stimulated cycle was 27.6% and in the artificial cycle was 24.7%. They detected that all three procedures were equally effective in pregnancy outcomes. Our results were similar to Konc *et al *(18). 

In a different study artificial cycle was compared with stimulated cycle which was induced by HMG for endometrial preparation prior to FET(19). There was no difference in clinical pregnancy rate (41.0 % vs. 41.6 %), ongoing pregnancy rate (36.6 % vs. 34.7 %) and live birth rate (30.0 % vs. 31.7 %) between two groups simillar to our study (19). Cochrane meta-analysis (2008) reported that there was no preference for methods to another (10). In a randomized multicenter clinical trial, Groenewoud* et al* in 2012 detected that there is no significant difference in live birth rates between natural and artificial methods (11). In another study in 2016, modified natural withthe artificial cycle for cryo-thawed embryo transferwere compared and it was observed observed that there is no significant differences in live birth rate (11.5% vs. 8.8%) and clinical pregnancy rate (19% vs. 16.0%) between two groups of modified NC-FET and AC-FET(12). A recent meta-analysis by Yarali* et al* shows that at the moment there is not sufficient evidence to support the use of any protocols (13).

## Conclusion

In the present study we found that there was no significant difference between letrozole protocol and artificial protocol for endometrial preparation in FET. Letrozole method has only one injection but in artificial method, daily injections should be done. 

## Note

***Registration ID in IRCT:*** ***IRCT201306256689N3***
